# Dynamic Parameters within the First 72 H of ICU Admission Predict Extubation Failure and 28‐Day Mortality in Severe Pneumonia‐Induced ARDS: A Retrospective Cohort Study

**DOI:** 10.1111/crj.70189

**Published:** 2026-04-23

**Authors:** Chen Wang, Yalong Liu, Wenqing Xu, Hanhan Hong

**Affiliations:** ^1^ Department of Respiratory and Critical Care Medicine, Changzheng Hospital Naval Medical University Shanghai China; ^2^ Department of Nursing, Changzheng Hospital Naval Medical University Shanghai China

**Keywords:** 72‐h dynamic parameters, acute respiratory distress syndrome, extubation failure, intensive care unit, mechanical ventilation, mortality, severe pneumonia

## Abstract

**Objective:**

To evaluate dynamic parameter changes within 72 h of intensive care unit (ICU) admission for predicting extubation failure and 28‐day mortality in patients with severe pneumonia‐induced acute respiratory distress syndrome (ARDS).

**Methods:**

This retrospective cohort study enrolled 424 adults with severe pneumonia‐induced ARDS receiving invasive ventilation ≥ 48 h during January 2023–August 2025. We collected clinical data and calculated 72‐h changes (Δ) in key parameters: physiological stress [maximum respiratory rate (RR), mean heart rate (HR)] and disease progression [ΔPaO_2_/FiO_2_, Δ blood lactate (BLA), Δ procalcitonin (PCT), Δ Sequential Organ Failure Assessment (SOFA)]. Outcomes were extubation failure (first spontaneous breathing trial failure or reintubation ≤ 48 h) and 28‐day mortality. Multivariable logistic and Cox regression models were built, with discrimination assessed by ROC curves.

**Results:**

Extubation failure and 28‐day mortality rates were 23.82% (101/424) and 29.48% (125/424), respectively. For extubation failure, independent risk factors included older age, higher APACHE II score at admission, immunosuppression, higher maximum RR, higher mean HR, and increased ΔBLA, ΔPCT, and ΔSOFA (protective factor: increased ΔPaO_2_/FiO_2_) (*p* < 0.05). The prediction model had an AUC of 0.912 (95% CI, 0.883–0.941). For 28‐day mortality, independent risk factors were higher APACHE II score at admission, higher maximum RR, increased ΔBLA, and increased ΔSOFA (*p* < 0.05), with a time‐dependent AUC of 0.755 (95% CI, 0.729–0.781). A significant association was observed between extubation failure and 28‐day mortality, with a markedly higher mortality rate in patients with extubation failure compared to those with successful extubation (81.19% vs. 13.31%, *p* < 0.001).

**Conclusion:**

Dynamic parameters within 72 h of ICU admission are predictors of extubation failure and 28‐day mortality in severe pneumonia‐induced ARDS, offering a tool for early risk stratification. Extubation failure was also strongly associated with increased short‐term mortality, underscoring its clinical significance as an adverse outcome during the disease course.

## Graphical abstract

Early 72‐h dynamic changes in key physiological and laboratory parameters predict extubation failure and 28‐day mortality in severe pneumonia‐induced ARDS. Extubation failure is strongly associated with increased mortality, highlighting the clinical value of dynamic monitoring for early risk stratification in ICU patients.

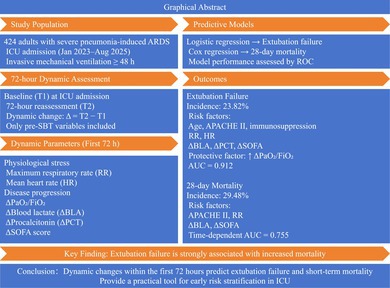

## 1 Introduction

Severe pneumonia is a leading and complex reason for intensive care unit (ICU) admission. Its tendency to progress rapidly to acute respiratory distress syndrome (ARDS) frequently necessitates invasive mechanical ventilation [1, 2, 3]. Although management strategies such as lung‐protective ventilation and prone positioning continue to advance, predicting which patients with severe pneumonia‐associated ARDS will wean successfully from the ventilator remains difficult. Weaning too early risks failed spontaneous breathing trials (SBT) or reintubation from insufficient alveolar recruitment, excessive respiratory muscle load, and unresolved circulatory or metabolic issues [4, 5]. Weaning too late, however, increases exposure to ventilator‐induced lung injury, ventilator‐associated pneumonia, sedation‐related complications, and diaphragmatic dysfunction, thereby prolonging mechanical ventilation and adversely affecting outcomes [6, 7]. Therefore, early and accurate identification of patients at high risk for weaning failure is a major clinical priority.

Conventional risk assessment often depends on static, single‐time‐point measurements, such as Acute Physiology and Chronic Health Evaluation (APACHE) II score, Sequential Organ Failure Assessment (SOFA) score, the ratio of arterial oxygen partial pressure to fractional inspired oxygen (PaO_2_/FiO_2_), lactate level, or infection markers at ICU admission [8, 9, 10]. While useful for baseline stratification, these snapshots do not capture the dynamic evolution of severe pneumonia‐induced ARDS. The crucial early ICU period, when responses to antibiotics and supportive care unfold, largely determines a patient's trajectory toward recovery or decline [11]. Relying solely on initial values can miss important trends; for example, two patients with similar baseline moderate ARDS may have opposite weaning outcomes if their oxygenation diverges over the next 72 h. Similarly, persistently high inflammatory and metabolic stress due to uncontrolled infection can lead to rapid decompensation post‐extubation, even if oxygenation shows transient improvement. A dynamic assessment of changes (Δ values) in key parameters may therefore better reflect the true clinical course and support real‐time decision‐making.

This study focuses on the first 72 h after ICU admission, a pivotal window for treatment response. We aim to evaluate whether dynamic changes in routinely measured physiological and laboratory parameters during this period can predict extubation failure and 28‐day mortality in ventilated patients with severe pneumonia‐induced ARDS. By shifting focus from static scores to dynamic trends, we seek to develop a practical bedside tool for early risk stratification, potentially guiding more individualized and timely clinical management.

## 2 Materials and Methods

### 2.1 Patient Population

This single‐center retrospective cohort study consecutively enrolled 424 patients admitted to the ICU of our hospital between January 2023 and August 2025. Inclusion criteria: Age ≥ 18 years; diagnosis of ARDS according to the Berlin criteria [12], with a confirmed etiology of severe pneumonia (community‐acquired or hospital‐acquired) [13]; treatment with invasive mechanical ventilation for ≥ 48 h; complete records of key physiological and laboratory parameters at ICU admission and within 72 h; first admission to the ICU in this study; survival for > 72 h after admission; complete clinical data allowing determination of primary outcomes (weaning status and 28‐day survival status). Exclusion criteria: ARDS due to noninfectious causes (severe trauma, aspiration, pancreatitis, burns, transfusion, etc.); chronic respiratory failure requiring long‐term home oxygen therapy or prior history of invasive mechanical ventilation; respiratory failure due to neurological disease (e.g., high spinal cord injury and myasthenia gravis); preexisting tracheostomy before ICU admission; coexistence of severe end‐stage diseases (e.g., New York Heart Association class IV heart failure, end‐stage liver disease, chronic kidney disease stage 5 requiring continuous renal replacement therapy, malignancy with expected survival < 3 months); withdrawal of life‐sustaining treatment within 72 h of ICU admission due to palliative care or decision to forego aggressive treatment; and missing key data or loss to follow‐up precluding outcome determination.

### 2.2 Data Collection

Patient data were retrospectively collected from the Hospital Information System (HIS) and Critical Care Clinical Information System (CCIS). Two trained researchers, blinded to the study outcomes, independently extracted and entered data. Cross‐checking was performed, with any discrepancies resolved by a senior investigator. Collected general clinical data included gender, age, body mass index (BMI), smoking history, alcohol history, comorbidities (hypertension, Type 2 diabetes mellitus, coronary atherosclerotic heart disease, chronic obstructive pulmonary disease, chronic kidney disease, chronic liver disease, etc., confirmed by medical record documentation), source of infection (categorized as community‐acquired or hospital‐acquired pneumonia), ARDS severity [classified as mild (200 mmHg < PaO_2_/FiO_2_ ≤ 300 mmHg), moderate (100 mmHg < PaO_2_/FiO_2_ ≤ 200 mmHg), or severe (PaO_2_/FiO_2_ ≤ 100 mmHg) based on the worst PaO_2_/FiO_2_ within 24 h of ICU admission per the Berlin criteria [12]], APACHE II score at ICU admission, immunosuppressive status [defined as any of human immunodeficiency virus infection; chemotherapy or radiotherapy within the past 6 months; long‐term use (> 2 weeks) of immunosuppressants (e.g., cyclosporine and tacrolimus) or prednisone equivalent dose ≥ 0.3 mg/kg/day; status post solid organ or hematopoietic stem cell transplantation], and pre‐ICU use of corticosteroids or empirical antibiotic therapy. To reflect parameter dynamics, key variables were characterized over the initial 72‐h period: (1) Physiological stress indicators: maximum respiratory rate (RR), mean heart rate (HR), and minimum mean arterial pressure (MAP); (2) disease progression indicators: calculated change values (Δ = value at 72 h after admission to the ICU − baseline value after admission to the ICU), including ΔPaO_2_/FiO_2_, Δ driving pressure, Δ blood lactate (ΔBLA), Δ procalcitonin (ΔPCT), and ΔSOFA score.

Time‐Window Definition: To minimize information leakage and reverse causality, all candidate predictors were restricted to objective monitoring and laboratory measurements obtained before the first weaning assessment/SBT. Dynamic indices (ΔPaO_2_/FiO_2_, ΔBLA, ΔPCT, and ΔSOFA) were defined as the change from the baseline value at ICU admission (T1) to the value at 72 h after admission (T2), calculated as Δ = T2 − T1. The T2 value was taken from the last routine assessment at the 72‐h time point (or the closest measurement within a window of 72 ± 5 h), provided it occurred prior to the first SBT initiation/extubation decision. Any measurements collected during the SBT, after the SBT outcome was known, or after extubation failure were excluded from the model.

### 2.3 Outcome Definitions

(1) Extubation Failure: According to the international consensus [14], extubation failure was defined as meeting either criterion: failure of the first SBT (using a T‐piece or low‐level pressure support ventilation, lasting 30–120 min), indicated by objective signs of respiratory, circulatory, or neurological deterioration; and requirement for reintubation due to respiratory failure within 48 h following planned extubation. Extubation success was defined as passing the SBT and remaining free from reintubation for 48‐h post‐extubation. The weaning assessment was carried out by the attending physician according to a unified procedure, including evaluations of sedation level, oxygenation and ventilation parameters, circulatory stability, and airway protection ability. The prediction model of this study was constructed only using the objective data obtained before the SBT to predict the outcome of weaning failure. The timing of weaning assessment and SBT was determined by the attending physicians based on standard clinical criteria, including improvement of the underlying disease, adequate oxygenation, hemodynamic stability, and sufficient level of consciousness. Although a generally consistent clinical approach was followed, minor variations in weaning practices may have existed due to the retrospective nature of the study.

(2) 28‐Day Mortality: The 28‐day mortality was defined as all‐cause death within 28 days after ICU admission. For patients discharged from the ICU before Day 28, follow‐up continued until 28 days after ICU admission to ascertain survival status. Survival status was verified through hospital records and telephone follow‐up when necessary.

### 2.4 Statistical Analysis

To ensure model stability and avoid overfitting, sample size was evaluated based on an events‐per‐variable (EPV) rule of thumb (≈10 events per predictor variable). For extubation failure (101 events, 23.82%), up to nine variables were included in the final multivariable model, yielding an EPV ≈ 11.2 (101/9). Given a failure rate of ~24%, a sample size of ~417 (100/0.24) was needed for ~100 events; the actual sample of 424 met this requirement. For 28‐day mortality (125 events, 29.48%), four variables entered the final Cox model, yielding an EPV ≈ 31.3 (125/4). With a mortality rate of ~29.5%, ~339 patients (100/0.295) were needed for ≥ 100 events; the sample of 424 also sufficed. Thus, sample size and event numbers supported the planned multivariable analyses and model construction.

SPSS 27.0 and R 4.2.1 software were used. Categorical data were presented as *n* (%) and compared using *χ* ^*2*^ or Fisher's exact test. Continuous data normality was assessed by Shapiro–Wilk test. Non‐normally distributed data were presented as M (P25, P75) and compared using Mann–Whitney *U* test. Normally distributed data were presented as $\bar{x}$ ± *s*, with comparisons using independent samples *t*‐test (if variance was homogeneous per Levene's test) or Welch's *t*‐test. Variables identified in univariate analysis (*p* < 0.05) underwent collinearity diagnosis [variance inflation factor (VIF) < 5]. Multivariable logistic regression (for extubation failure) and Cox proportional hazards regression (for 28‐day mortality) were employed to build prediction models. For the Cox regression model, a global test based on Schoenfeld residuals was used to evaluate its proportional hazards assumption (*p* > 0.05 indicates no violation). The receiver operating characteristic (ROC) curve was used to evaluate the model's discrimination, and the corrected values of the area under the ROC curve (AUC) and its 95% confidence interval (CI) were calculated using the bootstrap method (1000 repetitions) for internal validation. A two‐tailed *p*‐value < 0.05 was considered statistically significant.

## 3 Results

### 3.1 Baseline Characteristics of the Study Population

The baseline demographic and clinical characteristics of the study cohort are shown in Table 1. A total of 424 patients with severe pneumonia‐induced ARDS were included, with a median age of 64.00 (55.00, 72.00) years, and 62.74% were male. The majority of patients presented with moderate‐to‐severe ARDS, and community‐acquired infection was the predominant etiology.

**TABLE 1:** Baseline demographic and clinical characteristics of the study cohort.

Indicator	Overall (*n* = 424)
Gender	
Male	266 (62.74)
Female	158 (37.26)
Age (years)	64.00 (55.00, 72.00)
BMI (kg/m^2^)	23.80 (21.00, 26.20)
Smoking history	143 (33.73)
Alcohol history	107 (25.24)
Comorbidities	294 (69.34)
Source of infection	
Community‐acquired	293 (69.10)
Hospital‐acquired	131 (31.90)
ARDS severity	
Mild	62 (14.62)
Moderate	239 (56.37)
Severe	123 (29.01)
APACHE II score at admission	21.00 (17.00, 25.00)
Immunosuppressive status	94 (22.17)
Pre‐ICU corticosteroids	110 (25.94)
Empirical antibiotics	385 (90.80)
Maximum RR (breaths/min)	29.00 (25.00, 33.00)
Mean HR (beats/min)	93.62 ± 16.04
Minimum MAP (mmHg)	67.00 (60.00, 73.00)
ΔPaO_2_/FiO_2_ (mmHg)	14.83 ± 47.85
Δ driving pressure (cmH_2_O)	1.00 (−1.00, 2.00)
ΔBLA (mmol/L)	0.00 (−0.68, 0.60)
ΔPCT (ng/mL)	3.50 (−0.90, 8.33)
ΔSOFA score	1.00 (−1.00, 2.00)

Abbreviations: APACHE II, Acute Physiology and Chronic Health Evaluation II; ARDS, acute respiratory distress syndrome; BLA, blood lactate; BMI, body mass index; HR, heart rate; ICU, intensive care unit; MAP, mean arterial pressure; PaO_2_/FiO_2_, ratio of arterial oxygen partial pressure to fractional inspired oxygen; PCT, procalcitonin; RR, respiratory rate; SOFA, Sequential Organ Failure Assessment.

Comorbid conditions were common, affecting 69.34% of patients, and 22.17% were immunocompromised. The median APACHE II score at ICU admission was 21.00 (17.00, 25.00), reflecting a relatively high severity of illness. Additional physiological and laboratory parameters, including dynamic changes within the first 72 h, are detailed in Table 1.

### 3.2 Univariate Analysis for Extubation Failure

Among the 101 patients who experienced weaning failure, 65 (64.36%) failed the first SBT, and 36 (35.64%) underwent reintubation due to respiratory failure within 48 h after planned extubation. Univariate analysis showed the extubation failure group had significantly higher age, APACHE II score at ICU admission, proportion with immunosuppressive status, maximum RR, mean HR, ΔBLA, ΔPCT, and ΔSOFA score and significantly lower ΔPaO_2_/FiO_2_ compared to the extubation success group (*p* < 0.05; Table 2).

**TABLE 2:** Univariate analysis for extubation failure.

Indicator	Extubation failure group (*n* = 101)	Extubation success group (*n* = 323)	*χ* ^2^ */t/Z*	*p*
Gender			0.149	0.700
Male	65 (64.36)	201 (62.23)	—	—
Female	36 (35.64)	122 (37.77)	—	—
Age (years)	67.79 ± 10.16	61.82 ± 12.74	4.833	< 0.001
BMI (kg/m^2^)	23.38 ± 3.41	23.83 ± 3.60	1.092	0.276
Smoking history	38 (37.62)	105 (32.51)	0.901	0.343
Alcohol history	29 (28.71)	78 (24.15)	0.850	0.357
Comorbidities	73 (72.28)	221 (68.42)	0.538	0.463
Source of infection			2.811	0.094
Community‐acquired	63 (62.38)	230 (71.21)	—	—
Hospital‐acquired	38 (37.62)	93 (28.79)	—	—
ARDS severity			0.837	0.658
Mild	12 (11.88)	50 (15.48)	—	—
Moderate	58 (57.43)	181 (56.04)	—	—
Severe	31 (30.69)	92 (28.48)	—	—
APACHE II score at admission	22.00 (19.00, 27.00)	20.00 (16.00, 24.00)	3.900	< 0.001
Immunosuppressive status	31 (30.69)	63 (19.50)	5.582	0.018
Pre‐ICU corticosteroids	28 (27.72)	82 (25.39)	0.218	0.640
Empirical antibiotics	90 (89.11)	295 (91.33)	0.455	0.500
Maximum RR (breaths/min)	33.00 (29.00, 37.00)	28.00 (25.00, 33.00)	5.874	< 0.001
Mean HR (beats/min)	98.80 ± 16.24	92.01 ± 15.66	3.774	< 0.001
Minimum MAP (mmHg)	65.58 ± 9.10	67.25 ± 8.84	1.639	0.102
ΔPaO_2_/FiO_2_ (mmHg)	−17.31 ± 34.76	24.88 ± 46.99	9.729	< 0.001
Δ driving pressure (cmH_2_O)	1.00 (0.00, 2.00)	1.00 (−1.00, 2.00)	0.889	0.374
ΔBLA (mmol/L)	0.50 (−0.10, 1.20)	−0.20 (−0.80, 0.40)	6.384	< 0.001
ΔPCT (ng/mL)	6.90 (0.60, 9.70)	2.60 (−1.20, 7.25)	3.716	< 0.001
ΔSOFA score	2.00 (1.00, 4.00)	0.00 (−1.00, 2.00)	6.184	< 0.001

Abbreviations: APACHE II, Acute Physiology and Chronic Health Evaluation II; ARDS, acute respiratory distress syndrome; BLA, blood lactate; BMI, body mass index; HR, heart rate; ICU, intensive care unit; MAP, mean arterial pressure; PaO_2_/FiO_2_, ratio of arterial oxygen partial pressure to fractional inspired oxygen; PCT, procalcitonin; RR, respiratory rate; SOFA, Sequential Organ Failure Assessment.

### 3.3 Multivariable Logistic Regression Analysis for Extubation Failure

Collinearity diagnosis showed that all VIFs were below 3, indicating no significant multicollinearity. The likelihood ratio test of the overall model was statistically significant (*χ* ^*2*^ = 202.207, *p* < 0.001). Multivariable analysis identified older age (OR = 1.036, 95% CI 1.009–1.064), higher APACHE II score at ICU admission (OR = 1.113, 95% CI 1.050–1.179), presence of immunosuppression (OR = 2.212, 95% CI 1.082–4.520), higher maximum RR (OR = 1.131, 95% CI 1.071–1.196), higher mean HR (OR = 1.021, 95% CI 1.002–1.041), increased ΔBLA (OR = 2.383, 95% CI 1.670–3.402), increased ΔPCT (OR = 1.080, 95% CI 1.028–1.135), and increased ΔSOFA score (OR = 1.375, 95% CI 1.199–1.576) as independent risk factors for extubation failure. An increase in ΔPaO_2_/FiO_2_ was a protective factor (OR = 0.978, 95% CI 0.970–0.986). All *p*‐values were < 0.05. The Hosmer–Lemeshow goodness‐of‐fit test indicated good model calibration (*χ* ^*2*^ = 5.400, *p* = 0.714) (Table S1 and Table 3).

**TABLE 3:** Multivariable logistic regression analysis for extubation failure.

Variable	*β*	SE	Wald	*p*	OR (95% CI)
Age	0.036	0.013	6.951	0.008	1.036 (1.009–1.064)
APACHE II score at ICU admission	0.107	0.029	13.188	< 0.001	1.113 (1.050–1.179)
Immunosuppression (1)	0.794	0.365	4.737	0.030	2.212 (1.082–4.520)
Maximum RR	0.124	0.028	19.170	< 0.001	1.131 (1.071–1.196)
Mean HR	0.021	0.010	4.527	0.033	1.021 (1.002–1.041)
ΔPaO_2_/FiO_2_	−0.022	0.004	29.386	< 0.001	0.978 (0.970–0.986)
ΔBLA	0.869	0.182	22.876	< 0.001	2.383 (1.670–3.402)
ΔPCT	0.077	0.025	9.213	0.002	1.080 (1.028–1.135)
ΔSOFA	0.318	0.070	20.766	< 0.001	1.375 (1.199–1.576)

Abbreviations: APACHE II, Acute Physiology and Chronic Health Evaluation II; BLA, blood lactate; HR, heart rate; ICU, intensive care unit; PaO_2_/FiO_2_, ratio of arterial oxygen partial pressure to fractional inspired oxygen; PCT, procalcitonin; RR, respiratory rate; SOFA, Sequential Organ Failure Assessment.

### 3.4 Diagnostic Performance of the Multivariable Logistic Regression Model

**FIGURE 1:**
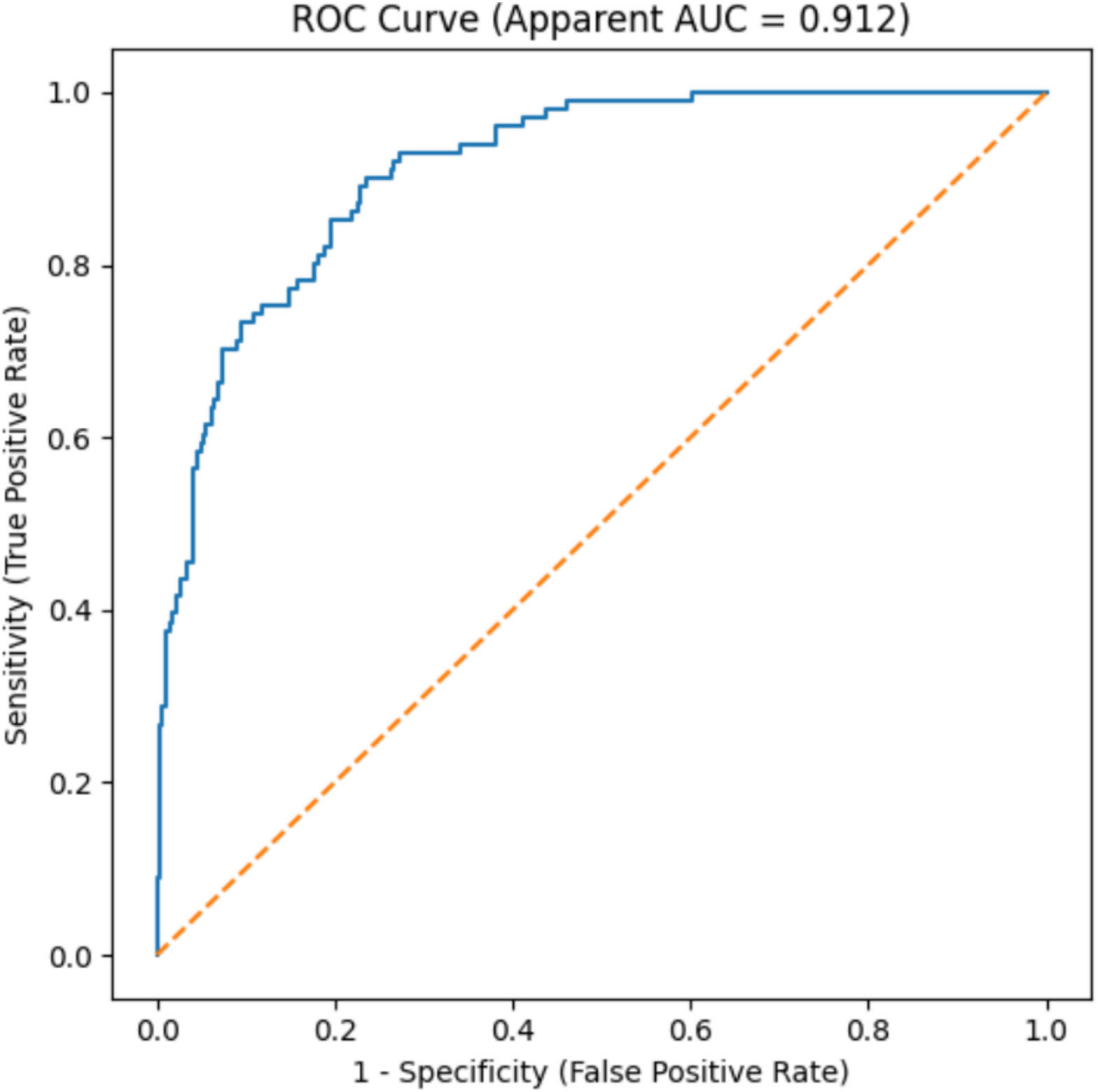
ROC curve of the logistic regression model for predicting extubation failure.

The ROC curve for the multivariable logistic regression model yielded an AUC of 0.912 (95% CI, 0.883–0.941), indicating excellent discrimination. After bootstrap internal validation (1000 repetitions), the optimism‐corrected AUC of the model was 0.900 (95% CI, 0.873–0.930), suggesting stable model performance. The maximum Youden's index (0.666) corresponded to sensitivity of 90.10% and specificity of 76.50% (Figure 1).

### 3.5 Univariate Analysis for 28‐Day Mortality

Univariate analysis showed that the deceased group had significantly higher age, APACHE II score at ICU admission, maximum RR, ΔBLA, ΔPCT, and ΔSOFA score and significantly lower ΔPaO_2_/FiO_2_ compared to survivors (*p* < 0.05; Table 4).

**TABLE 4:** Univariate analysis for 28‐day mortality.

Indicator	Deceased group (*n* = 125)	Survival group (*n* = 299)	*χ* ^2^ */t/Z*	*p*
Gender			1.511	0.219
Male	84 (67.20)	182 (60.87)	—	—
Female	41 (32.80)	117 (39.13)	—	—
Age (years)	65.90 ± 11.51	62.13 ± 12.65	2.873	0.004
BMI (kg/m^2^)	23.45 ± 3.60	23.83 ± 3.55	1.005	0.315
Smoking history	43 (34.40)	100 (33.44)	0.036	0.850
Alcohol history	31 (24.80)	76 (25.42)	0.018	0.894
Comorbidities	93 (74.40)	201 (67.22)	2.135	0.144
Source of infection			2.163	0.141
Community‐acquired	80 (64.00)	213 (71.24)	—	—
Hospital‐acquired	45 (36.00)	86 (28.76)	—	—
ARDS severity			0.109	0.947
Mild	19 (15.20)	43 (14.38)	—	—
Moderate	71 (56.80)	168 (56.19)	—	—
Severe	35 (28.00)	88 (29.43)	—	—
APACHE II score at admission	22.00 (17.00, 27.00)	20.00 (16.00, 24.00)	2.743	0.006
Immunosuppressive status	34 (27.20)	60 (20.07)	2.599	0.107
Pre‐ICU corticosteroids	39 (31.20)	71 (23.75)	2.549	0.110
Empirical antibiotics	109 (87.20)	276 (92.31)	2.753	0.097
Maximum RR (breaths/min)	32.00 (26.00, 36.00)	29.00 (25.00, 33.00)	3.799	< 0.001
Mean HR (beats/min)	95.71 ± 15.59	92.75 ± 16.17	1.736	0.083
Minimum MAP (mmHg)	67.00 (61.00, 73.00)	66.00 (60.00, 72.00)	0.896	0.370
ΔPaO_2_/FiO_2_ (mmHg)	1.30 ± 45.93	20.48 ± 47.58	3.823	< 0.001
Δ driving pressure (cmH_2_O)	1.00 (−1.00, 2.00)	1.00 (−1.00, 2.00)	0.183	0.855
ΔBLA (mmol/L)	0.42 ± 1.03	−0.17 ± 0.86	6.040	< 0.001
ΔPCT (ng/mL)	4.80 (−1.00, 9.50)	3.00 (−0.85, 7.50)	2.185	0.029
ΔSOFA score	2.00 (0.00, 4.00)	1.00 (−1.00, 2.00)	4.845	< 0.001

Abbreviations: APACHE II, Acute Physiology and Chronic Health Evaluation II; ARDS, acute respiratory distress syndrome; BLA, blood lactate; BMI, body mass index; HR, heart rate; ICU, intensive care unit; MAP, mean arterial pressure; PaO_2_/FiO_2_, ratio of arterial oxygen partial pressure to fractional inspired oxygen; PCT, procalcitonin; RR, respiratory rate; SOFA, Sequential Organ Failure Assessment.

### 3.6 Multivariable Cox Regression Analysis for 28‐Day All‐Cause Mortality

Collinearity diagnosis showed that all VIFs were < 3. The overall model omnibus test demonstrated statistical significance (*χ* ^*2*^ = 82.990, *p* < 0.001). For the Cox proportional hazards model, a global test based on Schoenfeld residuals showed no violation of the proportional hazards assumption (*p* > 0.05). After adjusting for covariates, higher APACHE II score at ICU admission (HR = 1.034, 95% CI 1.002–1.067), higher maximum RR (HR = 1.036, 95% CI 1.005–1.068), increased ΔBLA (HR = 1.431, 95% CI 1.201–1.705), and increased ΔSOFA score (HR = 1.148, 95% CI 1.071–1.232) were independent risk factors for 28‐day all‐cause mortality (*p* < 0.05). Age, ΔPaO_2_/FiO_2_, and ΔPCT did not show independent predictive value in the multivariable model (*p* > 0.05) (Table S2 and Table 5).

**TABLE 5:** Multivariable Cox regression analysis for 28‐day all‐cause mortality.

Variable	*β*	SE	Wald	*p*	HR (95% CI)
Age	0.010	0.007	1.914	0.167	1.010 (0.996–1.025)
APACHE II score at ICU admission	0.034	0.016	4.448	0.035	1.034 (1.002–1.067)
Maximum RR	0.035	0.015	5.283	0.022	1.036 (1.005–1.068)
ΔPaO_2_/FiO_2_	−0.003	0.002	2.299	0.129	0.997 (0.993–1.001)
ΔBLA	0.359	0.089	16.094	< 0.001	1.431 (1.201–1.705)
ΔPCT	0.021	0.013	2.361	0.124	1.021 (0.994–1.048)
ΔSOFA	0.138	0.036	15.008	< 0.001	1.148 (1.071–1.232)

Abbreviations: APACHE II, Acute Physiology and Chronic Health Evaluation II; BLA, blood lactate; ICU, intensive care unit; PaO_2_/FiO_2_, ratio of arterial oxygen partial pressure to fractional inspired oxygen; PCT, procalcitonin; RR, respiratory rate; SOFA, Sequential Organ Failure Assessment.

### 3.7 Diagnostic Performance of the Multivariable Cox Regression Model

**FIGURE 2:**
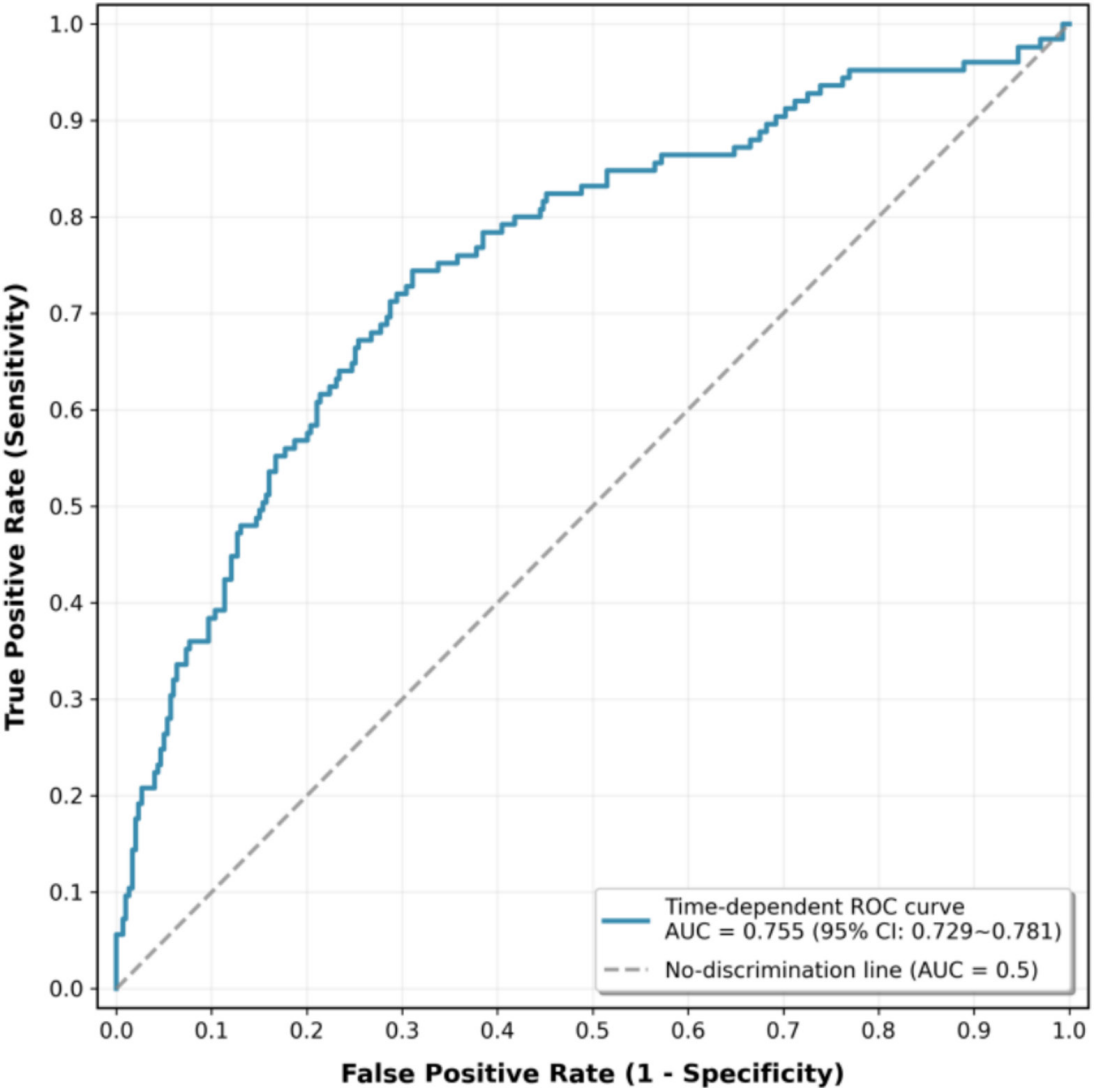
Time‐dependent ROC curve of the Cox proportional hazards model for predicting 28‐day all‐cause mortality.

The time‐dependent ROC curve for the final Cox regression model yielded an AUC of 0.755 (95% CI, 0.729–0.781) at the 28‐day time point, indicating moderate discrimination (Figure 2).

### 3.8 Association Between Extubation Failure and 28‐Day Mortality

A contingency table analysis showed a significant association between extubation failure and 28‐day mortality. Among the 101 patients with extubation failure, 83 died within 28 days, corresponding to a mortality rate of 82.18%, whereas among the 323 patients with successful extubation, 42 died within 28 days, corresponding to a mortality rate of 13.00%. The difference was statistically significant ($x^{2}$ = 177.096, *p* < 0.001). These findings indicate that extubation failure was strongly associated with an increased risk of 28‐day mortality (Table 6).

**TABLE 6:** Association between extubation failure and 28‐day mortality.

Extubation status	Death (*n* = 125)	Survival (*n* = 299)
Failure (*n* = 101)	83 (82.18%)	18 (17.82%)
Success (*n* = 323)	42 (13.00%)	281 (87.00%)
$x^{2}$	177.096	
*p*	< 0.001	

## 4 Discussion

This study, in a cohort of adults with severe pneumonia‐induced ARDS receiving invasive mechanical ventilation, found extubation failure and 28‐day all‐cause mortality rates of 23.82% (101/424) and 29.48% (125/424), respectively. These figures highlight the substantial ongoing risk in this population and the real‐world need for effective risk identification tools. Centered on the concept of dynamic changes within the first 72‐h post‐ICU admission, the findings are insightful from both pathophysiological and practical clinical perspectives. First, dynamic change values more directly reflect treatment response and disease trajectory; second, most included parameters are routinely available in the ICU, facilitating bedside application.

Regarding extubation failure prediction, multivariable logistic regression identified older age, higher APACHE II score at admission, immunosuppressive status, higher maximum RR, higher mean HR, increased ΔBLA, increased ΔPCT, and increased ΔSOFA as independent risk factors, whereas increased ΔPaO_2_/FiO_2_ was protective. The model demonstrated excellent discrimination (AUC = 0.912). These findings align with the pathophysiology of weaning failure. Older age and higher APACHE II score indicate poorer physiological reserve and greater overall severity, predisposing to respiratory workload imbalance and circulatory instability during weaning [15]. Immunosuppression suggests greater difficulty controlling infection, delayed resolution of inflammation, and higher risk of secondary infection, thereby impeding recovery of oxygenation and organ function [16]. Persistently elevated RR and HR reflect increased respiratory load and systemic stress, often indicating ventilation/perfusion mismatch, poor compliance, or elevated respiratory muscle load [17, 18]. An increase in ΔBLA suggests unresolved perfusion–metabolic stress. Patients may meet formal SBT criteria but remain vulnerable to rapid circulatory or metabolic decompensation post‐extubation [19]. Increased ΔPCT more strongly indicates persistent infectious burden or inadequate antimicrobial response, potentially sustaining inflammation‐driven capillary leakage and organ injury [20]. Increased ΔSOFA score summarizes multiorgan dysfunction evolving unfavorably, indicating systemic decompensation, which aligns closely with the systemic nature of weaning failure [21]. Conversely, increased ΔPaO_2_/FiO_2_ signifies improved oxygenation and reduced pulmonary inflammation/edema, forming the core physiological basis for successful weaning. Notably, Δ driving pressure did not show a stable independent association, suggesting that isolated respiratory mechanics changes may be insufficient to explain weaning outcomes, or its effect may be subsumed by more global variables such as oxygenation and organ function evolution. Future studies could incorporate more detailed ventilatory phenotypes such as mechanical power, patient–ventilator synchrony, and respiratory drive for validation.

For 28‐day mortality prediction, multivariable Cox regression identified higher APACHE II score at admission, higher maximum RR, increased ΔBLA, and increased ΔSOFA as independent risk factors. Age, ΔPaO_2_/FiO_2_, and ΔPCT lost independent predictive value after adjustment. The 28‐day time‐dependent AUC was 0.755, indicating moderate discrimination. This result suggests that short‐term mortality represents a composite outcome of systemic organ failure and perfusion–metabolic failure. APACHE II and ΔSOFA jointly characterize mortality risk from the perspectives of baseline severity and subsequent organ function evolution [22]. Persistently high RR serves as a signal of sustained high load, potentially reflecting uncontrolled pulmonary pathology, respiratory muscle fatigue, or a systemic hypermetabolic inflammatory state [23]. ΔBLA is a sensitive marker of inadequate tissue perfusion and metabolic disturbance [24]. The loss of independence for oxygenation and PCT dynamics in the multivariable model may imply that their influence on mortality is largely mediated through the organ failure pathway integrated by SOFA and lactate. This suggests that clinicians should not underestimate mortality risk based solely on transient improvements in oxygenation or declining infection markers, and close attention to lactate clearance and organ function trajectory is crucial to identify signals of ongoing systemic failure [25]. In addition, extubation failure was strongly associated with increased 28‐day mortality in our cohort. This finding suggests that extubation failure may serve as an important indicator of disease severity and adverse clinical progression. However, as it represents a downstream event during follow‐up, it should be interpreted as a marker of clinical deterioration rather than a baseline predictor.

From a clinical application standpoint, a strength of this study is the construction of two distinct outcome models based on a 72‐h dynamic window. The extubation failure model, with high discrimination, is more suitable as a risk assessment tool to inform weaning decisions. It can help identify patients undergoing weaning assessment or SBT who require a more cautious approach (e.g., delayed extubation, intensified secretion management and lung recruitment, optimization of analgesia/sedation and nutrition, evaluation of diaphragmatic function, planning for noninvasive ventilation/high‐flow nasal cannula transition, or earlier tracheostomy evaluation) [26, 27]. The mortality model, with moderate discrimination, is better suited for early prognosis communication and resource allocation, highlighting patients who may need more aggressive organ support and escalation of infection control (e.g., closer hemodynamic monitoring, lactate clearance‐guided resuscitation, rapid source control and antimicrobial re‐evaluation, and enhanced multidisciplinary consultation) [28]. Furthermore, using the consensus definition of extubation failure facilitates comparison with prior studies and potential translation into clinical quality metrics.

Of course, this study has several limitations. First, its single‐center retrospective design is susceptible to selection and information bias. Despite standardized data extraction and collinearity checks, unmeasured confounders (e.g., sedation depth, fluid balance, neuromuscular blocker use, exposure to prone positioning, weaning protocol variations, detailed ventilator settings, nutritional and rehabilitation interventions, and bacteriology and resistance profiles) could influence outcomes. Current clinical guidelines recommend that weaning readiness should be assessed based on a combination of respiratory, hemodynamic, and neurological criteria, including adequate oxygenation, stable cardiovascular status, and improvement of the underlying cause of respiratory failure [29]. However, due to the retrospective design, strict standardization of weaning protocols and detailed capture of SBT timing were not feasible. Second, dynamic parameters were captured only up to 72 h. While practical, this window may not reflect subsequent secondary insults (e.g., secondary infection, hemorrhage, arrhythmia, and worsening renal injury), which might partly explain the mortality model's lower discrimination compared to the extubation failure model. Third, the models currently demonstrate internal discrimination only, and external and prospective validation are lacking. Differences in patient populations, weaning practices, pathogen profiles, and treatment pathways across centers necessitate multicenter validation to assess generalizability. Fourth, the study used simple change values to characterize dynamics. While straightforward, this approach does not capture the shape of the trajectory (e.g., early rapid improvement followed by a plateau, continuous linear deterioration, and fluctuations). Future work could employ higher‐frequency time‐series analysis or machine learning to extract trajectory features, potentially improving prediction for complex outcomes such as mortality.

## 5 Conclusion

In summary, this study focuses on dynamic parameters within the first 72‐h post‐ICU admission in patients with severe pneumonia‐induced ARDS. It demonstrates the significant predictive value of early disease trajectory evolution for both extubation failure and 28‐day mortality. A well‐discriminating model for extubation failure and a moderately discriminating model for mortality are developed. The core contribution lies in transforming readily available bedside vital signs and routine laboratory values/scores from static descriptors into dynamic trend assessments, offering a risk stratification approach more aligned with real‐world ICU decision‐making logic. Future multicenter prospective external validation, coupled with the integration of additional information on respiratory mechanics, microbiology, and treatment exposures, holds promise for developing a generalizable dynamic risk prediction tool. Such a tool could ultimately help reduce extubation failure rates, optimize resource allocation, and improve short‐term survival outcomes in patients with severe pneumonia‐induced ARDS.

## Author Contributions

**Chen Wang:** conceptualization, data curation, investigation, validation, writing – original draft, writing – review and editing. **Yalong Liu:** data curation, formal analysis, investigation, validation, writing – original draft, writing – review and editing. **Wenqing Xu:** data curation, formal analysis, software, visualization, writing – original draft, writing – review and editing. **Hanhan Hong:** conceptualization, methodology, project administration, resources, supervision, writing – review and editing. All authors have read and approved the final version of the manuscript.

## Funding

The authors have nothing to report.

## Ethics Statement

This retrospective study was approved by the Ethics Committee of Changzheng Hospital, Naval Medical University (Approval No. 2023SL007) and conducted in accordance with the Declaration of Helsinki. All data were anonymized and handled confidentially.

## Conflicts of Interest

The authors declare no conflicts of interest.

## Supporting information


**Data S1:** Supporting Information.

## Data Availability

The data used and/or analyzed during the current study are available from the corresponding author.

## References

[1] L. Papazian , M. Klompas , and C. E. Luyt , “Ventilator‐Associated Pneumonia in Adults: A Narrative Review,” Intensive Care Medicine 46, no. 5 (2020): 888–906, 10.1007/s00134-020-05980-0. 32157357 PMC7095206

[2] M. S. Niederman and A. Torres , “Severe Community‐Acquired Pneumonia,” European Respiratory Review 31, no. 166 (2022): 220123, 10.1183/16000617.0123-2022. 36517046 PMC9879347

[3] D. Adriao , F. Mojoli , R. Gregorio Hernandez , D. De Luca , B. Bouhemad , and S. Mongodi , “Ventilator‐Associated Pneumonia: An Update on the Role of Lung Ultrasound in Adult, Pediatric, and Neonatal ICU Practice,” Therapeutic Advances in Pulmonary and Critical Care Medicine 20 (2025): 29768675251349632, 10.1177/29768675251349632. 40528864 PMC12171069

[4] Y. J. Chen , Y. F. Zhang , D. L. Huo , D. Luo , and W. W. Chen , “Risk Factors and Nomogram for Predicting Mechanical Ventilation in Severe Pneumonia,” Frontiers in Medicine 12 (2025): 1598952, 10.3389/fmed.2025.1598952. 41030261 PMC12477012

[5] P. Ochoa , A. R. Mendoza , D. Molano , J. R. Masclans , and H. M. Parada‐Gereda , “Risk Factors and Outcomes of Ventilator‐Associated Pneumonia: An Updated Systematic Review and Meta‐Analysis,” BMC Pulmonary Medicine 25, no. 1 (2025): 453, 10.1186/s12890-025-03932-2. 41053703 PMC12502355

[6] K. J. Park , “Lung‐Protective Ventilation Strategy in Acute Respiratory Distress Syndrome: A Critical Reappraisal of Current Practice,” Critical Care 29, no. 1 (2025): 444, 10.1186/s13054-025-05675-2. 41121191 PMC12538788

[7] H. Wu and B. Chasteen , “Rapid Review of Ventilator‐Induced Diaphragm Dysfunction,” Respirology 223 (2024): 107541, 10.1016/j.rmed.2024.107541. 38290603

[8] Y. Wang , G. Chen , M. Han , et al., “Early Lactate Trajectories Predict Mortality in Sepsis‐Associated Acute Lung Injury: A Retrospective Cohort Study,” Respiratory Medicine 249 (2025): 108471, 10.1016/j.rmed.2025.108471. 41192742

[9] Y. Deng , S. Li , J. Li , et al., “Enhancing Mortality Prediction in Intensive Care Units: Improving APACHE II, SOFA, and SAPS II Scoring Systems Using Long Short‐Term Memory,” Internal and Emergency Medicine 20, no. 8 (2025): 2541–2550, 10.1007/s11739-025-03896-5. 40325281

[10] R. Tan , C. Ge , Z. Li , et al., “Early Prediction of Mortality Risk in Acute Respiratory Distress Syndrome: Systematic Review and Meta‐Analysis,” Journal of Medical Internet Research 27 (2025): e70537, 10.2196/70537. 40392588 PMC12134695

[11] X. Shi , Y. Shi , L. Fan , et al., “Prognostic Value of Oxygen Saturation Index Trajectory Phenotypes on ICU Mortality in Mechanically Ventilated Patients: A Multi‐Database Retrospective Cohort Study,” Journal of Intensive Care 11, no. 1 (2023): 59, 10.1186/s40560-023-00707-x. 38031107 PMC10685672

[12] L. Papazian , C. Aubron , L. Brochard , et al., “Formal Guidelines: Management of Acute Respiratory Distress Syndrome,” Annals of Intensive Care 9, no. 1 (2019): 69, 10.1186/s13613-019-0540-9. 31197492 PMC6565761

[13] J. P. Metlay , G. W. Waterer , A. C. Long , et al., “Diagnosis and Treatment of Adults With Community‐Acquired Pneumonia. An Official Clinical Practice Guideline of the American Thoracic Society and Infectious Diseases Society of America,” American Journal of Respiratory and Critical Care Medicine 200, no. 7 (2019): e45–e67, 10.1164/rccm.201908-1581ST. 31573350 PMC6812437

[14] T. D. Girard , W. Alhazzani , J. P. Kress , et al., “An Official American Thoracic Society/American College of Chest Physicians Clinical Practice Guideline: Liberation From Mechanical Ventilation in Critically Ill Adults. Rehabilitation Protocols, Ventilator Liberation Protocols, and Cuff Leak Tests,” American Journal of Respiratory and Critical Care Medicine 195, no. 1 (2017): 120–133, 10.1164/rccm.201610-2075ST. 27762595

[15] L. Zhou , P. Zhou , C. Gao , T. Li , and Q. Zhou , “Real‐Time Predictive Analysis of ICU Ventilator Weaning Failure: A Prospective Validation Study,” Clinical Respiratory Journal 19, no. 11 (2025): e70136, 10.1111/crj.70136. 41188069 PMC12585916

[16] S. Arora , S. M. Walker , K. W. Cummings , and M. M. Hammer , “Pneumonia in Immunocompromised Patients,” Radiographics 45, no. 12 (2025): e250021, 10.1148/rg.250021. 41296608

[17] F. Sterr , M. Reintke , L. Bauernfeind , et al., “Predictors of Weaning Failure in Ventilated Intensive Care Patients: A Systematic Evidence Map,” Critical Care 28, no. 1 (2024): 366, 10.1186/s13054-024-05135-3. 39533438 PMC11556093

[18] J. Menguy , K. De Longeaux , L. Bodenes , B. Hourmant , and E. L'Her , “Defining Predictors for Successful Mechanical Ventilation Weaning, Using a Data‐Mining Process and Artificial Intelligence,” Scientific Reports 13, no. 1 (2023): 20483, 10.1038/s41598-023-47452-7. 37993526 PMC10665387

[19] X. Ruan , X. Zhang , W. Hu , L. Wu , and Y. Shen , “Risk Factors for Failed Extubation Within 7 Days in Elderly Critically Ill Patients Based on Respiratory Mechanics and Clinical Indicators: A Retrospective Cohort Study,” Frontiers in Medicine 12 (2025): 1721952, 10.3389/fmed.2025.1721952. 41346987 PMC12672513

[20] R. H. Elshaboury , J. P. Metlay , M. Broyles , C. Rhee , E. J. Giamarellos‐Bourboulis , and M. K. Mansour , “Procalcitonin in the Management of Lower Respiratory Tract Infection and Sepsis,” Journal of Antimicrobial Chemotherapy 81, no. 1 (2026): dkaf298, 10.1093/jac/dkaf298. 41312720 PMC12957930

[21] R. Wang , B. Qi , X. Zhang , L. Meng , and X. Wu , “Prophetic Values of Lung Ultrasound Score on Post‐Extubation Distress in Patients With Acute Respiratory Distress Syndrome,” European Journal of Medical Research 27, no. 1 (2022): 27, 10.1186/s40001-022-00652-9. 35193686 PMC8864851

[22] M. T. Beigmohammadi , L. Amoozadeh , F. Rezaei Motlagh , et al., “Mortality Predictive Value of APACHE II and SOFA Scores in COVID‐19 Patients in the Intensive Care Unit,” Canadian Respiratory Journal 2022 (2022): 5129314, 10.1155/2022/5129314. 35356088 PMC8958381

[23] M. Li , F. Liu , Y. Yang , et al., “Identifying Vital Sign Trajectories to Predict 28‐Day Mortality of Critically Ill Elderly Patients With Acute Respiratory Distress Syndrome,” Respiratory Research 25, no. 1 (2024): 8, 10.1186/s12931-023-02643-8. 38178157 PMC10765902

[24] Y. Yang , Y. Wang , G. Zhu , S. Xu , J. Liu , and Z. Tang , “Developing a Predictive Nomogram for Mortality in Patients With Extrapulmonary Acute Respiratory Distress Syndrome: The Prognostic Value of Serum Soluble Thrombomodulin, Lung Ultrasound Score, and Lactate,” Frontiers in Pharmacology 15 (2024): 1407825, 10.3389/fphar.2024.1407825. 39257391 PMC11385278

[25] R. Diab , R. Bou Chebl , N. Barmo , et al., “Prognostic Utility of Procalcitonin and Lactate Clearance for In‐Hospital Mortality in Sepsis,” Frontiers in Medicine 12 (2025): 1679297, 10.3389/fmed.2025.1679297. 41357484 PMC12675444

[26] K. J. Roberts , L. T. Goodfellow , C. M. Battey‐Muse , et al., “AARC Clinical Practice Guideline: Spontaneous Breathing Trials for Liberation From Adult Mechanical Ventilation,” Respiratory Care 69, no. 7 (2024): 891–901, 10.4187/respcare.11735. 38443142 PMC11285503

[27] S. M. Fernando , A. Tran , B. Sadeghirad , et al., “Noninvasive Respiratory Support Following Extubation in Critically Ill Adults: A Systematic Review and Network Meta‐Analysis,” Intensive Care Medicine 48, no. 2 (2022): 137–147, 10.1007/s00134-021-06581-1. 34825256

[28] L. Evans , A. Rhodes , W. Alhazzani , et al., “Surviving Sepsis Campaign: International Guidelines for Management of Sepsis and Septic Shock 2021,” Intensive Care Medicine 47, no. 11 (2021): 1181–1247, 10.1007/s00134-021-06506-y. 34599691 PMC8486643

[29] J. M. Boles , J. Bion , A. Connors , et al., “Weaning From Mechanical Ventilation,” European Respiratory Journal 29, no. 5 (2007): 1033–1056, 10.1183/09031936.00010206. 17470624

