# Supplementary material for: Dynamic Parameters within the First 72 H of ICU Admission Predict Extubation Failure and 28‐Day Mortality in Severe Pneumonia‐Induced ARDS: A Retrospective Cohort Study
Source: Clin Respir J. 2026 Apr 23;20(5):e70189. doi: 10.1111/crj.70189 (PMC13106213; doi:10.1111/crj.70189)
Supplement: Supplementary file 1 — Data S1: Supporting Information. [file CRJ-20-e70189-s001.docx]

Supplementary Table S1 Variable assignment for logistic regression

| Variable | Factor | Assignment |
| --- | --- | --- |
| X1 | Age | Continuous |
| X2 | APACHE II score at ICU admission | Continuous |
| X3 | Immunosuppression | No = 0, Yes = 1 |
| X4 | Maximum RR | Continuous |
| X5 | Mean HR | Continuous |
| X6 | ΔPaO_2_/FiO_2_ | Continuous |
| X7 | ΔBLA | Continuous |
| X8 | ΔPCT | Continuous |
| X9 | ΔSOFA | Continuous |

Note: APACHE II: Acute Physiology and Chronic Health Evaluation II; ICU: Intensive care unit; RR: Respiratory rate; HR: Heart rate; PaO_2_/FiO_2_: Ratio of arterial oxygen partial pressure to fractional inspired oxygen; BLA: Blood lactate; PCT: Procalcitonin; SOFA: Sequential Organ Failure Assessment.

Supplementary Table S2 Variable assignment for Cox regression

| Variable | Factor | Assignment |
| --- | --- | --- |
| X1 | Age | Continuous |
| X2 | APACHE II score at ICU admission | Continuous |
| X3 | Maximum RR | Continuous |
| X4 | ΔPaO_2_/FiO_2_ | Continuous |
| X5 | ΔBLA | Continuous |
| X6 | ΔPCT | Continuous |
| X7 | ΔSOFA | Continuous |

Note: APACHE II: Acute Physiology and Chronic Health Evaluation II; ICU: Intensive care unit; RR: Respiratory rate; PaO_2_/FiO_2_: Ratio of arterial oxygen partial pressure to fractional inspired oxygen; BLA: Blood lactate; PCT: Procalcitonin; SOFA: Sequential Organ Failure Assessment.
